# First time description of dismantling phenomenon

**DOI:** 10.3389/fpsyg.2015.00510

**Published:** 2015-05-05

**Authors:** Laurence Barrer, Guy Gimenez

**Affiliations:** Laboratoire Psychologie Clinique Langage et Subjectivité (EA 3278), Départment de Psychologie, UFR de Lettres et Sciences Humaines, Aix Marseille Université, Aix-en-Provence, France

**Keywords:** countertransference, autistic disorder, dismantling, metapsychology, defense mechanism

## Abstract

Dismantling is a complex psychic phenomenon, which is not easy to define, and little interest has been shown in the subject. The authors of this paper want to demonstrate that dismantling is the main defense mechanism in autism, bringing about de-consensus of senses. The effects perceived in a child with autistic disorder are passivity and lack of thought. The authors’ purpose here is to define the dismantled state and reveal its underlying process. This paper will therefore describe for the first time in literature, the dismantling phenomenon and will submit a metapsychological approach of this defense mechanism.

The psychic dismantling phenomenon, which we name as dismantled state, has not been fully described in scientific literature. Meltzer (1972) first used the term to describe a process in autism characterized by an immediate and transitory suspension of mental activity to explain and develop his concept, he first suggested a possible link between, what he termed as “the dismantled object” and two external real objects: the transitional object and the fetish object. He made a connection between the dismantled object and the sexual object used in perversions (the fetish). However, Meltzer abandons this work path and then in 1975 compares dismantling to obsessional mechanisms. He adds that the compulsive repetition would be a dynamic element of the dismantling process, a way of controlling objects and bringing them together (Meltzer, 1975, p. 32–34). Their conjunction would occur on the basis of their “essential function” (Meltzer, 1975, p. 270) which is to dismantle the experience into “a condition of simpleness beneath the level of ’common sense”’ and “to find aimless mechanical interlinkage between its parts.” He associated this to the bizarre objects of Bion (1957) which are psychotic objects fragmented into minute parts after splitting. These particles created are ejected through projection out of the Ego and thus become raw and unprocessed. Nevertheless, this conceptualization was incomplete. Among existing research on autism, we will focus here on the work referring to psychoanalytic paradigm.

## The Dismantling Concept

In 1975, a theoretical overhaul leads Meltzer to develop ideas such as de-consensus of senses, absence of pain as an autistic process and the staggering reaction of the child with autistic disorder faced with the beauty of an aesthetic object. He also draws to light the love the child with autistic disorder has for the preservation of the maternal shelter (Meltzer, 1986, p. 237). When he uses his three comparisons, he mentions the idea of the existence of a general organizer [the cement of the wall for the first one (Meltzer, 1975, p. 30), the strings of the toy dog actioned by someone for the second one (Meltzer, 1975, p. 31) and the commanding captain for the third (Meltzer, 1975, p. 31)] but he does not develop this idea further and does not refer to it throughout his later work. Meltzer specifies that a child with autism under dismantling process is without thought and that “time spent in autism is lost for maturation” (Meltzer, 1975, p. 30). Meltzer (1986) referred to Bion’s (1974) work on thinking activity by linking the dismantling process to the concept of truth, which would be at the origin of the psychic growth and development. He defines the dismantling process as an activity of destruction of thoughts leading to falsity.

In summary, dismantling is a psychic movement which is a process of sensorial de-consensus where senses roam “to the most attractive object of the moment” (Meltzer, 1975, p. 31). He later describes dismantling as a state of great passivity causing the autistic child to suspend his attention—and considers it a passive process. However, he builds his concept from literary comparisons without exemplifying with clinical material taken from children with autistic disorder. Today, Meltzer’s literary comparisons are systematically reused by authors studying autism (Boubli, 2002; Ribas, 2002; Houzel, 2006; Golse and Delion, 2008; Hochmann, 2009; Golse, 2011) and we find few clinical descriptions of the dismantling phenomenon (Hoxter, 1975; Lheureux-Davidsé and Barabé, 2005), although Meltzer (1986, p. 144) has opened a field of research and instigated the scientific community to explore further.

Meltzer (1986) imagines the dismantling process as a mental capacity, which would shield off pain and psychic conflict and to allow defense against reality but nowhere did he refer to it as a defense mechanism. For this author, the etiology of dismantling is not in the infant-mother’s inter-subjectivity. On the contrary, he supports the idea that the child’s personality, his extreme sensitivity, his high intelligence and his strong emotional potential when meeting other people are at the origin of the use of the dismantling process. He introduces the conception of Ego dismantling as a deconstruction, a sudden dislocation of the senses, which were previously linked by a consensual real-life experience.

To further explain the dismantling phenomenon, Ciccone and Lhopital (1991), Meltzer’s successors, refer to the action of the death drive in autism: it’s action would consist of the creation of a never-ending psychical inward withdrawal similar to what Houzel (1985) described as a whirling spiral downward movement. They furthermore described dismantling as a defense mechanism. Dismantling would be a defense mechanism against primitive anxiety related to the death drive. They named it defense mechanism as did Soulé et al. (1985) which Meltzer (1972, 1975, 1986) did not. On the other hand, other authors such as Haag (2005, 2006), assumed that dismantling is due “to overwhelming emotion when hearing a voice and/or a penetrating look which seem to cut off and dissociate the development of the sensory information from their perceptive organization (which is permitted by attentional function) and to seriously damage the cognitive development.” To contribute to the understanding of this phenomenon, we lead research on children with autistic disorder.

## Description of Dismantling Phenomenon

Our research was based upon data collected over 2 years of clinical and therapeutic sessions. One twenty seven videos were taken and each one was analyzed. In order that our clinical material followed a logical sequence with our research hypotheses, we needed to lead our investigations in a scientific dimension. To do so, we had to define general standards of proof to test our hypotheses (Bazan, 2011). To identify these standards of proof, we used the IPA qualitative method of clinical observation (Interpretative Phenomenological Analysis, Smith and Osborn, 2003).

In the videos, our patients adopted similar attitudes when dismantling and we tried to list in details the attitudes, signs and movements that occurred simultaneously. We assembled the clinical data and distinguished five standards of proof:

(1) The stare at a unique steady point called “attraction point” recognizable by the discontinuity of its colors, light and texture;
(2) The muscle tone, either low or high according to the therapeutic progress;
(3) The attention focused on precise dots which have no interconnection;
(4) Temporarily suspended movements except for a repetitive auto-sensual movement of a visible part of the body (hand, arm…) or a non-visible part (larynx, sphincter, respiratory system…);
(5) The sudden and immediate capacity to enter or extract oneself of this dismantled state.

When a dismantled state occurs, the child with autistic disorder starts staring. The palpebral reflex is slowed or stopped. This look gives the impression that the child with autistic disorder is elsewhere, that he is no longer seeing. It is often referred to as a “blank stare” in literature. We pointed out that the stare is focused on an “attraction point” characterized by the discontinuity of colors (blue object on red surface…) of light (a ray of light in a dark room, the incandescent wire filament of a bulb), of contrast (a crumb on a dark floor, sequins on the black dress…), of texture (something rough on a smooth or soft surface).

We first made the assumption that a hypotonic posture would be found during the dismantling phenomenon but we unexpectedly observed a systematic reversal of the muscle tone. During our research (Barrer, 2013), the general muscle tone of the child with autistic disorder was changed to hypotonic when hypertonic in everyday life and to hypertonic when hypotonic. However, we noticed that the muscle tone changed to hypertonic only after a certain period of therapy. At the beginning of our research, the children usually showed hypotonic posture during the dismantling phenomenon.

The attention of the child with autistic disorder is not reduced or lacking but in fact has no junctional aim. Attention seems focused on non-connected precise points of the environment. This scattered attention gives the impression that the subject has left his immediate environment, that he is elsewhere, disconnected from reality, atonic, lifeless.

When a dismantled state occurs, all activity is suspended whether the muscle tone is hypo- or hypertonic. We have however, discovered that a part of the child’s body, like a finger, a hand or a leg, remains in action or is activated. That movement is rhythmical and auto sensual although the body is atonic; it can be visible (hand, foot…) or invisible (sounds from the larynx, self satisfactory feelings of vibrating frictions). The reason for this movement seems to be to maintain an anchorage in reality. Atony is low muscle tone with non-reactive deep-tendon reflexes. On the contrary, hypertonicity is the abnormal raising of muscle tone. When extracted from the hypertonic dismantling state, the autistic child starts to concentrate, collects himself a short moment around the discovery of others (touching and looking at him). Hypertonicity could be at the origin of the gradual opening up.

The dismantled state appears in seconds (according to the measures taken on the video films). The transition between the moment where the child is active (playing, walking, watching...) and the moment where he is dismantled is not gradual but sudden.

In our research, we tried to demonstrate that each sense is focalised on precise sensorial points and to show that these are not linked, as Meltzer (1975) reported. The sensorial unlinking gives the observer the impression that the child is closed off from his environment however, the fact that his attention is scattered between each of his senses shows he is present, but only on these sensorial points. In this state, the autistic child stops thinking and is not totally present. This unlinked presence leads to passivity; he cannot filter what is heard, smelt, tasted and touched as if he had regressed to an archaic mode where senses have not yet been linked, thanks to the gathering experience brought by the mothering object.

## Conception of Dismantling as Defense Mechanism

The following definition, inspired by S. Freud and A. Freud has provided a basis for us: defense mechanisms are “different types of processes through which defense may be given specific expression. Which of these mechanisms predominate in a given case depends upon the type of illness under consideration, upon the developmental stage reached, upon the extent to which the defensive conflict has been worked out, and so on. It is generally agreed that the ego puts defense mechanism to use, but the theoretical question whether their mobilization always presupposes the existence of an organized ego capable of sustaining them is an open one” (Laplanche and Pontalis, 1967).

The understanding of defense mechanisms is essential to help patients using other adaptive strategies than the ones, which is cutting themselves off from the others and from themselves. The description of dismantling as a defense mechanism allows the practitioner to identify it more easily during sessions and therefore treated it more efficiently. Our approach suggests that patient’s symptoms are the result of ego’s attempt to deal with a painful psychic conflict, it’s like he could deal at that time: as if he could do so in that specific moment. Therapy’s purpose would be then helpful to the Subject who find in therapeutic link, other resources to help them deal with the conflict, in a more adaptive way which would also reduce psychic suffering.

The psychic process consists of devitalizing the link to the other. In a previous research (Barrer, 2013) it was pointed out that the dismantling process attacks a part of the perceptive reality, which seems to be perceived as an “attracting object”, i.e., an attracting object toward a consensual experience (Bick, 1968). But instead of evacuating that part of reality bearing a conflict, it is the experience itself that is transformed by the child with autistic disorder into a sensorial flow. This assigns the attracting object to be a mere thing and is no longer a possible subject of a relationship. Affect is repelled and, as Barthélémy and Gimenez (2011) wrote, “the reification of the affect” places the other in “a dimension of concretism” or “an agglomerate of sensorial perceptions.” Dismantling can be considered as an active defense mechanism whose task is to unlink sensations (Bion, 1959) arising from the various senses, leaving the consensual function (Anzieu, 1985) inefficient.

The child then lives a physical experience through several sensorial canals. The I concentrate each sensory canal and kinesthetic perception on one experience, which is focused on a specific sensorial point, different for each sensory canal. On that specific sensorial point this focus separate (isolation defense) the I from the perceptions that come from the other sensory canals. These experiences build what we term as a dismantled Ego where pictogramic (Aulagnier, 1975) traces of each sensation exist but separate (unlinked, delinked) from any other similar ones.

Another repercussion of the dismantling process is the annihilation of thoughts by a triple caesura of the links (Barrer, 2013) between: the primal pictogramic traces issued from one and the same experience; these traces and their potential representations (which could at a certain point contain the thought and give a meaning to it); between these traces and the investment directed toward them. Death drive seems to be, for us, what lies at the source of the dismantling process. Its expression is revealed intrapsychically, by the destruction of the psychic bonds, dislocating the child’s senses, and physically with hypotony as the expression of the return to non-organic matter.

Dismantling, as a psychic and active process, is at the origin of the incapacity to initiate a relationship. We have noticed that a child with autistic disorder massively dismantles when he is in a location which could possibly cause him to meet an Other (Barrer, 2013).

## Metapsychological Model

We have started to develop a metapsychological model of the dismantling process in autistic disorder. Meltzer (1975) had outlined the features of this model.

From a dynamic point of view, the dismantling process is an active process used as a defense mechanism. During the dismantling process, the conflict between death and life drives appears through the swing from passive posture during dismantling to re-activation. The child swings from de-consensuality to re-consensuality. This mechanism offers the possibility to fight against the awareness of the other’s presence, the Other being a potential attracting object permitting the formation of a bond. During the dismantled state, the various senses are delinked one from another.

During dismantling phenomenon, the function of the perceptual apparatus is altered. Each sense organ provides a sensation, which is kept independent from the other, monosensorial sensation (pictogramic representation). These sensations are not coupled with the modalities of perception (their is no interaction permitting the creation of the meaning of the reality): the conjunction of sense-data fails, therefore common sense is affected (Bion, 1967). Anzieu (1985, p. 127) says that common sense “which the baseline is always relative to the sense of touch” is altered and the “intersensorial function” of the skin-ego is attacked. The child cannot experiment the situation he is living because this situation is de-consensualited and reduced to a simple perception, only sensory and proprioceptive (Segal, 2009). The I is momentarily anaesthetized (Poupard et al., 2004): what he experiences is just an apposition of sensations which are not linked to their inner perceptions. The sense of reality disappears leaving the child in unreadable dimension.

From a structural point of view we can refer to Meltzer’s (1975) work in which he suggests that the underlying conflict in autism is between “the Id, the Ego and the ideal Ego” (p. 29). We draw a parallel between this “ideal Ego” and Klein’s (1945) early superego and Freud’s (1914, 1923) ideal Ego (*idealich*), the ideal of omnipotent narcissistic power. We underline the fact that the entities present in dismantled state are the Id, with its death drives (hetero- or auto-aggression, destruction or self-destruction) and life drives (sexual and self-preservative) on one hand and the ideal ego on the other hand. Dismantling process provoques a triple attack against link. The fist happens between the primal pictogramic inscriptions of a self-experience (Gimenez, 2000). The second takes place between these pictograms located in the Unconscious and the possible word representations which could have contained the experience and give sense to it. The third, between the imprint of the pictogram and the investment directed toward them. There is no symbolisation of the sensorial self-experience during dismantled state but it exists as pre-symbolic pictogramic traces.

From an economic point of view, during the dismantling process, the quantity of energy flow (the quota of affect) is scattered on each sense which creates a partitioning of the energy charge and hence a reduction of the tension. Each sense being focalized on a pictogram (punctic concentration describe by Bion, 1959), the affect is then neither linked nor free but just deposited in each of the objects that the sensations are centered on (monosensoriel way). Dismantling helps the child to extract himself from the outer world and from a relationship to the other. It has a function: an autistic protection. It produces disinvestment of the world around, reality is suspended, to over invest it element per element, creating pictograms simultaneously. Libido is free and is redirected to the only possible reality, the pictograms in the *hic et nunc*. We believe that the autistic shell (Tustin, 1981) is built according to that function of the dismantling process. By unlinking the self-experiences in sensuous forms, the child with autistic disorder has no conscious representative of either what he is experiencing or of with whom. This creates an autistic envelope called “a shell.”

From a developmental point of view, the dismantling process is the reviviscence of the de-consensual state the infant lives through during his first months after birth when he has not yet experienced the consensual state supported by the capacity of a third party to combine senses. The child with autistic disorder uses as he wishes this de-consensual state where the mother-object is stopped in its consensualizing function. Interactions with the environment do not combine and remain separate, preventing stable object relations to be elaborated. As for the genetic point of view, Meltzer (1975) assumed that the occurrence of the dismantling process depended on the consensualizing capacity of the mother-object. The infant uses that process during the growing dyadic infant/mother relationship, when the attraction of the mother-object creates an aesthetic shock causing the withdrawal from the relationship. The sensorial consensualizing process, the remanteling, re-gathering process cannot take place. Meltzer (1975) considers that the origin of the dismantling is the impossible access to consensual attraction, resulting in the suspension of the attention toward the outer world. The child is like suspended, dismantled. Our hypothesis differs in that point, we suggest that during the dismantled state, the child is awaiting the mother-object, and this state exists not only in the autistic condition in children.

From a therapeutic point of view, we suggest that this analysis of the dismantled state can help the therapist when working with children with autistic disorder. The therapist can help the re-gathering of senses, remantling, by offering re-consensualizing experiences through the transference process. Directly soliciting the child with autistic disorder can lead him to invest sensorial experiences consensualized by an Other. This complex situation for the therapist has lead us to describe a therapeutic process based on pictograms (Barrer, 2013), a way of behaving according with remantling process and associated with an evolution of the transference—counter transference process.

## Conclusion

Throughout this article we have described the dismantling process and studied its underlying defense mechanism as a major defense mechanism in autistic disorder. We have also tried to contribute to the elaboration of a metapsychological approach of Meltzer’s (1975) concept.

Our clinical experience with children with autistic disorder lead us to question ourselves on the dismantling process. Our research also lead us to describe the phenomenon, the aim of which being to model that process. We have proposed a modeling of the dismantling process and are convinced that a theoretical and psychopathological field is open to other autistic phenomena revealed in the 80^′^s.

### Conflict of Interest Statement

The authors declare that the research was conducted in the absence of any commercial or financial relationships that could be construed as a potential conflict of interest.
